# Essential Oils Encapsulated in Zeolite Structures as Delivery Systems (EODS): An Overview

**DOI:** 10.3390/molecules27238525

**Published:** 2022-12-03

**Authors:** Alexandra P. Ferreira, Cristina Almeida-Aguiar, Susana P. G. Costa, Isabel C. Neves

**Affiliations:** 1 CQUM—Centre of Chemistry, Chemistry Department, University of Minho, 4710-057 Braga, Portugal; 2 CBMA—Centre of Molecular and Environmental Biology, Department of Biology, University of Minho, 4710-057 Braga, Portugal; 3 CEB—Centre of Biological Engineering, University of Minho, 4710-057 Braga, Portugal; 4 LABBELS—Associate Laboratory, 4710-057 Braga, Guimarães, Portugal

**Keywords:** essential oils, zeolites, encapsulation, delivery system, antimicrobial activity

## Abstract

Essential oils (EO) obtained from plants have proven industrial applications in the manufacturing of perfumes and cosmetics, in the production and flavoring of foods and beverages, as therapeutic agents in aromatherapy, and as the active principles or excipients of medicines and pharmaceutics due to their olfactory, physical-chemical, and biological characteristics. On behalf of the new paradigm of a more natural and sustainable lifestyle, EO are rather appealing due to their physical, chemical, and physiological actions in human beings. However, EO are unstable and susceptible to degradation or loss. To tackle this aspect, the encapsulation of EO in microporous structures as zeolites is an attractive solution, since these host materials are cheap and non-toxic to biological environments. This overview provides basic information regarding essential oils, including their recognized benefits and functional properties. Current progress regarding EO encapsulation in zeolite structures is also discussed, highlighting some representative examples of essential oil delivery systems (EODS) based on zeolites for healthcare applications or aromatherapy.

## 1. Introduction

**Figure 1:**
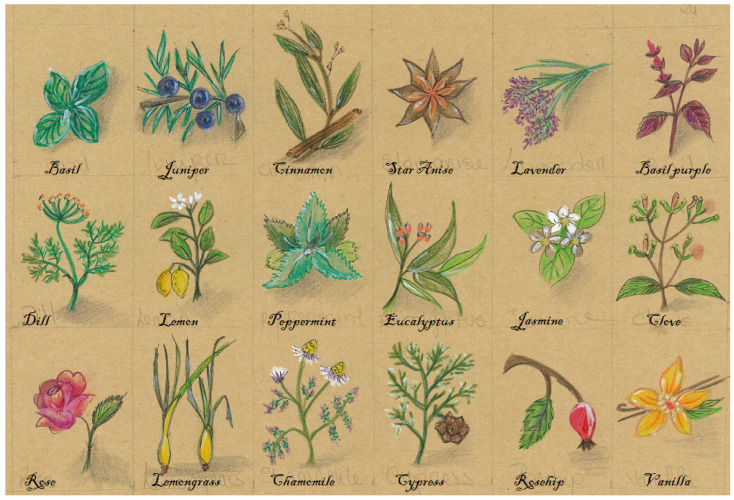
Representation of different EO-producing aromatic plants (author ICN).

Essential oils (EO) are volatile secondary metabolites produced by aromatic plants and characterized by an intense odor. These natural substances are mixtures of fragrant substances or mixtures of fragrant and odorless substances that are liquid and soluble in both organic solvents and lipids [1,2]. Among these aromatic plants, basil (*Ocimum basilicum*), lemongrass (*Cymbopogon* spp.), vanilla (*Vanilla* spp.), and cinnamon (*Cinnamomum verum*), among others (Figure 1), are the sources of several specific and very interesting EO with different properties.

These complex mixtures can contain over 300 different compounds, usually with molecular weights lower than 300 Da [3,4]. The compositions and relative proportions of the components are not only dependent on intrinsic factors, such as metabolism, the plant growth stage, or which part of the plant is used to obtain the EO, but also on extrinsic factors, such as the extraction method, solvent used, and environmental conditions [1,2]. Generally, the larger compounds define the biologic and biophysical properties of an essential oil, the magnitude of its effects being dependent on the concentration [1]. 

**Figure 2:**
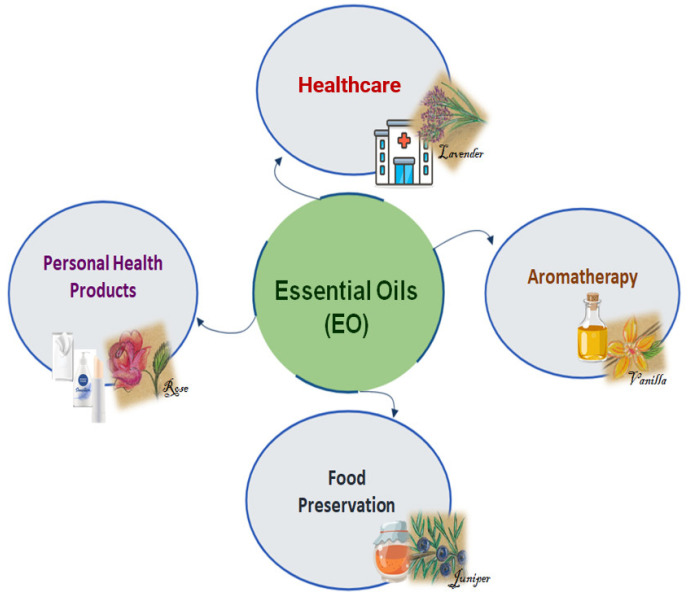
Some EO applications.

Several EO show very promising biological applications, given the absence of toxicity at very low concentrations, such as in perfumes, personal care products, food preservation, and in medicine [5] (Figure 2).

EO-based aromatherapies are subject to an increasing demand for therapeutic applications in hospital environments due to their positive physical and physiological actions in patients. The most widely used EO in aromatherapy programs are sweet orange (*Citrus sinensis*), lavender (*Lavandula angustifolia*), roman chamomile (*Chamaemelum nobile*), peppermint (*Mentha x piperita*), ginger root (*Zingiber officinale*), and mandarin (*Citrus reticulate*), among others [6]. As these compounds are physically and chemically unstable and susceptible to degradation or loss, encapsulating such EO in porous materials—as essential oil delivery systems (EODS)—is an attractive way to preserve their actions and properties. Since EO are very volatile, encapsulation offers the ability to preserve the molecules’ integrity against degradation and allows for a controlled release, provided that the host material has such characteristics [7].

Zeolites used as host materials offer a stable crystalline structure with channels, cavities, and nanopores in a defined microporous arrangement (sub-nanometer to 2 nm), with a high surface area and acid/thermal stability. These microporous materials are very cheap and have found applications in several domains, including detergents, adsorbents, and catalysis [8,9,10,11]. Moreover, the low toxicity of these structures in biological environments enables their use in the biomedical field as antimicrobial materials [12,13], drug delivery systems [14], and theragnostic applications [15].

This work reviews the fundamental information regarding essential oils, including their widely recognized benefits and functional properties. Subsequently, the current progress of EO encapsulation in zeolite structures is discussed, presented, and described with representative examples. In this review, we endeavor to evaluate the zeolites’ capacity for EO encapsulation, thus paving the way for the design of better essential oil delivery systems (EODS) for food, textiles, and healthcare applications.

## 2. Essential Oils (EO)

**Figure 3:**
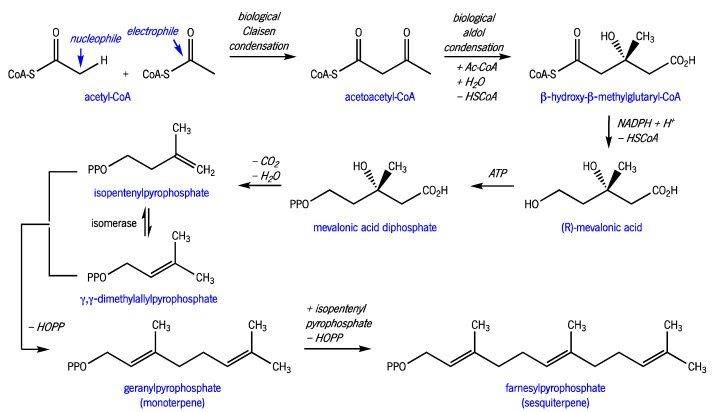
Monoterpene and sesquiterpene biosynthetic pathways. (CoA, coenzyme A; NADPH, reduced form of nicotinamide adenine dinucleotide phosphate; ATP, adenosine triphosphate; HOPP, hydroxy-pyrophosphate; PP, -P(=O)(OH)-O-P(=O)(OH)_2_ (adapted from [17]).).

**Figure 4:**
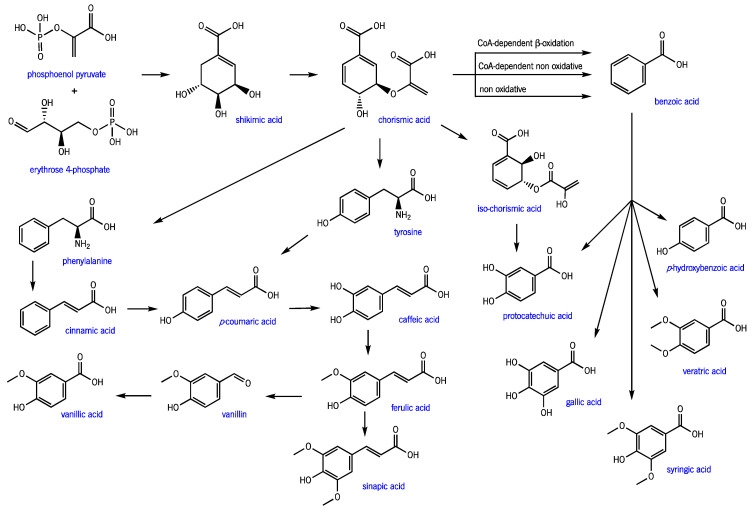
Shikimate pathway for phenolic compound biosynthesis in plants (adapted from [19]).

EO are present in specialized cells or glands of various organs of some plants. Acting as chemical signals, they allow the plant to attract pollinator insects, repel predators, inhibit seed germination, and perform allopathic communication [3,16,17,18]. Terpenes and terpenoids, biosynthesized via the mevalonate pathway (Figure 3), are the most abundant compounds in EO. Phenolic compounds, derived from the shikimate pathway (Figure 4), are also present but typically appear in vestigial quantities. All these compounds are responsible for the olfactory, physicochemical, and biological characteristics of the complex EO space. 

EO components are considered to be intracellular mediators of oxidation, protecting the host from the actions of atmospheric agents [20,21]. Additionally, they are a source of energy in conditions of deficient carbon assimilation. Although present in plant-specific tissues, they influence perspiration and other important functions, such as the retardation of nyctinasty, seismonastia, phototropism, and geotropic movements. EO can also inhibit the formation of chlorophyll in etiolated plants and reduce the permeability of cellular membranes [20,21]. EO play an important role in plant protection, particularly as antibacterial, antifungal, and antiviral agents, as insecticides, and in reducing the plant appetites of herbivores [1]. Some components, such as menthol, ascaridole, or methyl eugenol, exhibit activity against pathogenic bacteria and fungi and also against pest insects [20,21]. 

Essential oils are primarily used as food aromas, cosmetic fragrances, and as drugs due to their functional properties (Table 1). In cosmetics, EO are used due to their antioxidant, deodorant, anti-inflammatory, antimicrobial, emollient, and humectant properties [22]. The antioxidant activity of EO is also very important for the preservation of food against the toxic effects of oxidation [16,23]. Additionally, the capacity for eliminating free radicals can be explored in the prevention of diseases resulting from cellular damage, such as cancer, cardiac disease, impaired immune system, and cerebral dysfunction. Anti-inflammatory and antioxidant properties are related, because an oxidative burst is an inflammatory response [16]. 

The antimicrobial activity of EO is dependent on their main compounds; however minor compounds can also modulate this activity and define the fragrance, density, established interactions, penetration, and/or the cellular distribution [31]. The distribution of EO inside the cell defines the reactions that produce free radicals [1,31]. 

### 2.1. Biologic Activity and Mechanisms of Action of EO as Antimicrobial Agents

Essential oils do not target specific cells, and bioactive compounds have the ability to adhere to the cell wall [31]. The inherent hydrophobicity enables their passage through the cell wall and cytoplasmic membrane, modifying structure of the cells and permeabilizing them [18]. The membrane’s integrity is disturbed by the accumulation of compounds, which influences the metabolism and leads to cell death. Therefore, EO antimicrobial activity involves a series of reactions that affect the whole cell [31]. Yet, the mechanism of action of an EO depends not only on the micro-organism but also on the EO itself [32] (Table 2). In bacteria, membrane permeabilization leads to ion loss, membrane potential reduction, the collapse of the proton bomb, and a reduction in ATP production [1,32]. Gram-negative bacteria are generally less susceptible than Gram-positive bacteria, which is explained by the presence of an external complex membrane that is rich in lipopolysaccharides and limits the diffusion of hydrophobic compounds [31,32]. Gram-positive bacteria have a thick peptidoglycan wall that cannot restrict access to the cellular membrane. The penetration of hydrophobic compounds is further facilitated by the presence of the lipophilic extremities of lipoteichoic acid in the cytoplasmic membrane [31,32].

In eukaryotic cells, mitochondrial membrane depolarization occurs due to a decrease in the membrane potential, the disturbance of ionic channels, and the reduction in the pH gradient. The membrane fluidity variation leads to the loss of radicals, cytochrome c, ions, and proteins, culminating in bioenergetic failure and cellular oxidative stress [1]. In filamentous fungi and yeast, the inhibition of ergosterol biosynthesis, disruption of the cytoplasmic membrane and mitochondrial integrity, and the loss of cellular contents, such as DNA, were demonstrated [31,33] (Table S1). Hyphae disintegration occurs due to the presence of monoterpenes and sesquiterpenes [34]. Enzymes involved in cell wall synthesis are damaged, changing the typical fungal morphology [34,35]. 

Microbial resistance is not broadly verified, since EO are composed of various components having different and specific mechanisms of action [36]. However, resistance or microbial adaptation has been detected, which can result from the non-specific activity of EO, continuous exposure to sub-lethal concentrations, or when micro-organisms have an intrinsic resistance to an individual EO component [1,36]. 

The prolonged use of sub-MIC (minimum inhibitory concentration) concentrations of EO can influence the susceptibility not only to these mixtures but also to antibiotics [36]. A four-fold MIC increase against *Morganella morganii* and *Proteus mirabilis* was verified when these bacteria were passed fifty times in *Origanum vulgare* oil [37]. *P. mirabilis* also demonstrated a progressive increase in its susceptibility to ampicillin, specifically showing a MIC minimum value of 64 mg/L to 8 mg/L after fifty passages in *Origanum vulgare* oil. However, when *Pseudomonas aeruginosa* and *Serratia marcescens* were exposed to the same conditions, no effect was observed. Moreover, no effects were detected when these four species were exposed to cinnamon oil under the same experimental conditions [37]. Although studies involving the repetitive exposure of micro-organisms to EO revealed an increase in the MIC values, such increases were significantly smaller than those verified using conventional antibiotics in similar setups [36]. 

The growth of *Bacillus cereus* in a sublethal concentration of carvacrol, a phenolic monoterpene, showed a reduction in the membrane fluidity as a result of the alterations in the ratio and composition of the fatty acids [1]. In *Pseudomonas aeruginosa*, the increased tolerance to *Melaleuca alternifolia* EO was associated with alterations in the external membrane composition and changes in the energetic function [1]. *Pseudomonas* spp. revealed tolerance to tea tree oil and its main components by expressing the MexAB-OprM efflux pump, indicating that is possible to develop an effective defense mechanism [36]. However, when there is a large cellular target, such as a membrane, or a higher number of components with distinct mechanisms of action, the development of resistance can be demanding and unbeneficial to the micro-organism [36]. The micro-organism may be annihilated or completely inhibited, even before developing methods of evasion to avoid the EO activity [27]. 

Crucial factors for EO activity are the composition and functional groups of the active compounds and their interactions [35,38,39]. Interactions between the numerous compounds of EO can possibly generate additive effects, in which the total activity is equivalent to the sum of the individual activity of each compound, be it synergistic, in which the value obtained is higher than the sum of the individual activities, or antagonistic, where the antimicrobial activity is lower than the sum of the effects of the individual components [38,40,41,42] (Table S2). 

The use of isolated components enables a constant chemical composition and known interactions [24]. The studies of Bassolé have shown a high synergy when combining eugenol with linalool or menthol, suggesting that the junction of a phenolic monoterpene with an alcoholic monoterpene is efficient [43]. The most efficient combinations of isolated EO components against *Listeria innocua* have been shown to be carvacrol and thymol, or carvacrol, thymol, and eugenol [44]. Yet, the EO antimicrobial activity is generally higher than the activity displayed by the mixtures of their principal components, a reflection of the synergistic effects of minor compounds [43,44]. Synergic, addictive, or antagonistic interactions can occur not only between compounds of the same EO but also between compounds of different EO or with other antimicrobial agents [40,41,45] (Table 3). For example, the interaction of mint EO, a weak antimicrobial essential oil, with *Pangamia pinnata* EO amplifies its activity [46]. The synergic properties of *Eucalyptus globulus* and *Laurus nobilis* or *Salvia officinalis* EO against Gram-positive bacteria enabled reductions 20 to 200 times those achieved with the use of synthetic preservatives [47]. Yet, the addition of methyl-*p*-hydroxybenzoate to the abovementioned EO combinations eliminates resistant micro-organisms, such as *Pseudomonas aeruginosa* [47].

The combination of EO and synthetic antibiotics/antifungals demonstrated many beneficial effects (Table S3), although, occasionally, some disadvantageous effects were also shown [32,42]. A two- to three-fold increase in the MIC (minimum inhibitory concentration) of tetracycline and nalidixic acid when *Serratia marcescens* was passed in oregano oil was reported [37]. An increase in the chloramphenicol, minocycline, and ciprofloxacin MICs was also verified but less noticeable. Additionally, an antagonistic effect against *Escherichia coli* was observed when *Eugenia uniflora* EO was combined with gentamicin [51].

### 2.2. Economic Viability of Essential Oils

EO extraction methods are diverse and may include the use of liquid carbon dioxide, microwaves, or high- or low-pressure distillation with water vapor or hot water [1]. The resulting chemical constitution varies not only in the component numbers but also in the stereoisomers displayed. For the purpose of exploiting EO antimicrobial properties, the most frequently used extraction method is vapor distillation, where water vapor passes through the plant tissues and destroys the olefin layer, volatilizing the EO [18]. Extraction with lipophilic solvents and, sometimes supercritical carbon dioxide, is commonly used in the perfume industry due to the hydrophobicity of EO [2,18]. Since EO constitute less than 1% of the plant contents, their extraction can be expensive, considering the high quantity of raw material required. Despite this, EO are effective against micro-organisms at reduced quantities [18].

The preservation of EO bioactivities also depends on the production process and conservation conditions. Conservation or storage should be performed in hermetically closed resistant glass and dark containers at temperatures varying between 15 and 20 °C. In optimal conditions, EO can be stored for up to three years [18].

When using natural compounds, attention should be given to the possible reduction in the antimicrobial activity with the dilution, pH change, volatilization, and absorption caused by the containers [52]. The use of plant extracts may be associated with changes in color, odor, and emulsion stability [32,53]. Some EO suffer color changes as well. For example, *Matricaria chamomilla* EO changes from blue to brown upon light exposure. Adulteration, incorrect extraction methods, contamination, and/or extended storage generate low-quality EO, affecting their olfactory, physical-chemical, and biological characteristics [54]. Encapsulation emerges as a viable solution to the limitations associated with the use of essential oils, since it restricts the influence of environmental variations, allows for a controlled release under the appropriate conditions, and amplifies EO stability [31,35,55,56]. The enclosed environment may hamper the conversion of the molecules by cyclization, isomerization, or oxidation, thus allowing the maintenance of the characteristics and specific activity of the EO [2,35] as an essential oil delivery system (EODS).

## 3. Zeolites as Carriers for the Encapsulation of Essential Oils

**Figure 5:**
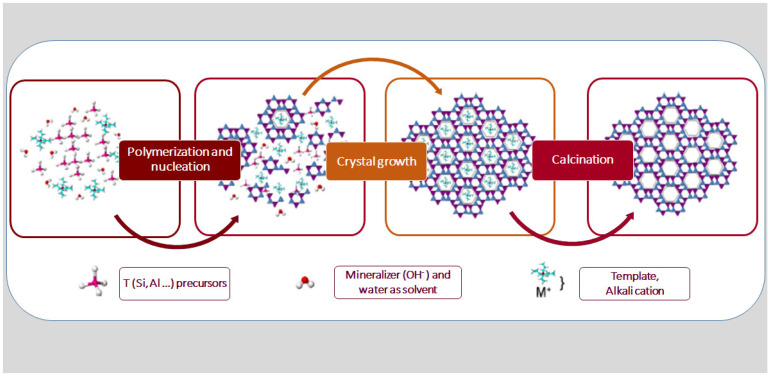
General scheme of zeolite synthesis (adapted from [59]).

Essential oil delivery systems (EODS) based on zeolites are very promising due to the unique properties of such structures. Zeolites are inorganic, solid materials composed of silicon (Si), aluminum (Al), and oxygen (O) organized in a 3D structure [57,58]. All zeolite frameworks can be built by linking a basic building unit (BBU), the tetrahedron TO_4_, with more complex building units, such as SiO_4_ and AlO_4_ tetrahedrons, in a periodic pattern, forming secondary building units (SBU) (Figure S1) [9,57]. Zeolite synthesis starts with the polymerization and nucleation of the precursor species, followed by crystal growth and calcination (Figure 5), to enhance the structure’s channels and pores [59]. The delimited range of the pore diameters classifies zeolites into small structures (LTA, *Linde type A* structure) of around 4 Å, medium (MFI, *Mobil type five* structure) structures of approximately 5.5 Å, and large (FAU, *faujasite* structure) structures with pores of 7.5 Å, according to the International Zeolite Association (IZA), which assigned three letter codes to over 40 natural and 230 synthetic zeolite frameworks [60,61].

Natural zeolites are typically formed in alkaline environments from volcanic sediments and materials. Hence, variations in the chemical composition are common, as well as irregular and defective structures [62,63,64,65]. Synthetic zeolites can be manufactured by various known approaches, such as hydrothermal synthesis, microwave-assisted hydrothermal synthesis, and solvothermal and ionothermal synthesis [62]. The hydrothermal synthesis method is considered the most dependable due to its low energy consumption, high reactivity, suitable condense phase, and simple solution control [62]. The resulting zeolites display a higher diversity of Si/Al ratios (usually, an Si/Al ratio of 1:1 in synthetic zeolites, in contrast with a 5:1 ratio in natural zeolites) and can contain a wider variety of framework elements that are not restricted to the Zn, Ge, P, B, Ga, and Be elements [64,66]. Nonetheless, their structure formation is affected, i.e., by the batch and reactant composition, water content, temperature, inorganic cations, and solvents [62].

The arrangement of the constituents of oxygen rings provides uniformly distributed pores and cavities with a peculiar arrangement, resulting in specific selectivity, ion exchange properties, and absorption capacity [62,63,67]. The differences in the structure, morphology, hydrophobic/hydrophilic properties, and chemical composition of natural and synthetic zeolites are reflected in their adsorption ability [56,68,69]. Since synthetic zeolites are often in the form of sodium or ammonium salts, monovalent/multivalent cations or metallic species can easily replace those cations, conferring specific absorption or catalytic properties [62]. Usually, zeolites with a low ratio of Si/Al present a greater ion exchange capacity, as well as a more hydrophilic character. However, not only are they susceptible to degradation in mild acid conditions, but they may also degrade and loose crystallinity at high temperatures in the presence of water [58,62,66]. The adsorption capability can be further amplified by the use of different methods, such as acid or heat activation [56]. Additionally, certain post-synthesis modifications allow for changes in the crystal size, porosity, and pore access [70]. 

The number of aluminum ions (Al^3+^) in the structure defines various important parameters of such aluminosilicates. A pure silica structure (SiO_2_) is neutrally charged. However, substituting the trivalent silicon with aluminum produces an aluminosilicate and changes the structure so that it becomes negatively charged [57]. Electroneutrality is maintained by compensating for the negative charge induced by Al^3+^ with interchangeable cations [57,62,71]. It also provides the structure with hydrophobicity and acidity, because the ion’s negative charge attracts (polar) water molecules [57]. 

The structural cavities and channels contain water molecules that create hydration spheres around the interchangeable cations [10,67]. The removal of water molecules at 350–400 °C enables the penetration of small molecules. The removal of, or change in, the cations in the structure can be achieved with cationic solutions due to their ion exchange capacity [10,11,67].

The structure of micropores scales from nanometers to sub-nanometers, with a high specific surface area, lack of toxicity, low cost, thermal stability, and chemical inertness, enabling the application of zeolites to many areas, such as environmental protection systems, drug delivery systems, sensors, absorption processes, catalysts, antimicrobial agents, and magnetic resonance imaging systems [9,10,11,12,14,15,56,61,64,72,73]. The widespread application of zeolites is mainly due to their inorganic-based framework of solid acid and ion-exchange efficiencies, as well their molecular sieving applications [64] (Table S4). Although zeolites are found in nature, synthetic zeolites represent 70% of those available on the global market [9].

Some zeolites have been given special attention due to their intrinsic antimicrobial or insecticidal activities [61]. Clinoptonite, a natural zeolite, is widely used in biomedical, environmental, and food applications [61]. Simultaneously, this zeolite, with its therapeutic, anti-inflammatory, antiproliferative, and pro-apoptotic properties, is the only zeolite registered in the EU as a medical product that can be used in oral treatment [61,72]. Khojaewa and colleagues (2019) showed that clinoptolite, chabazite, and natrolite reduced the viability of colorectal cancer cells by 30, 40, and 60%, respectively, suggesting a possible therapeutic application in the treatment of malignant neoplasms [73]. An experimental assay was conducted to evaluate the insecticidal activity of diatomaceous earth, three natural zeolites, and a synthetic zeolite (Type 4A) against *Sitophilus zeamais*, a major pest affecting stored maize and other cereals [66]. The diatomaceous earth demonstrated the highest insecticidal activity, followed by the natural zeolites, whose activity was associated with a higher content of silicon dioxide when compared with the synthetic zeolite [66]. Further environmental, food, and medical applications of zeolites were extensively revised by Derakhshankhah [64] and Bacakova [74].

Zeolites can be modified through the introduction of compounds possessing antimicrobial properties [74], including metal ions such as silver. As an antimicrobial agent, silver displays a wide spectrum of actions, high thermal stability, low volatility, and inertia [12,13,58]. Their high ion exchange capacity makes zeolites ideal for the accumulation of one or more metals in negatively charged sites. Simultaneously, it is possible to develop antimicrobial agents, since the release of metal ions is slow, and the regeneration occurs through secondary ionic change [12]. Antimicrobial properties depend on the zeolite’s pore size, physical appearance, and the nature of the metal used [58]. The mode of action of zeolites with encapsulated silver affects the release of metal ions in the solution, cellular water loss, cell entry, and inhibition of DNA replication. The environmental accumulation, antimicrobial activity, and cytotoxicity to animal cells are dependent on the concentration [12], release profile, and degree of silver oxidation [12,58]. Additionally, the silver ion is easily reduced to metallic silver, which can decrease the activity or require the use of special treatment to avoid it [13,58]. Moreover, its incorporation in transport systems is efficient in terms of the cost when compared with the direct use of silver [12].

Zeolites of Linde type A (LTA) embedded with silver demonstrated antibacterial activity against *Bacillus subtilis*, *Escherichia coli*, and *Staphylococcus aureus* [75]. The encapsulation of silver in zeolites of type X (FAU) also displayed bactericidal effects against Pseudomonas aeruginosa, *Escherichia coli*, and *Staphylococcus aureus* [13,58]. Other metals, such as copper and zinc, have been used to produce antimicrobial zeolites, but silver still shows a higher antibacterial activity [58]. Copper, a cheaper and more stable metal than silver, is used in sterilizing solutions, textiles and dental materials [12]. The mechanism of action is not fully established; however, it is believed that it results from the metallic cation entry into the cell and the subsequent cellular membrane damage due to the formation of reactive oxygen species (ROS) [12]. The antimicrobial and anti-inflammatory properties of zinc ions allow for its application in hospital contexts [12,76]. The positive charges of metallic species interact electrostatically with negatively charged cellular membranes, interfering with their permeability [12]. A greater inhibition of fungal growth was reported for zinc- and copper-based zeolites in contrast to silver zeolites [58]. This effect could be related to the fact that zinc and copper are essential elements for fungi, becoming toxic in concentrations that exceed the micro-organism’s requirements [58].

The combination of EO with silver nanoparticles (SNP) amplifies their antimicrobial activity through their combined mechanisms of action against diverse pathogenic and multi-resistant agents [31], allegedly through summative or synergic interactions. Cinnamaldehyde, the major compound of cinnamon EO, demonstrated a high synergy with SNP against the spore-forming Gram-positive bacteria *Bacillus cereus* and *Clostridium perfringens*, inducing a quick bactericidal action and extensive cellular membrane damage [31,77]. *Cymbopogon* EO showed synergy with silver cations (Ag^+^) against *Staphylococcus aureus*, *Enterococcus faecalis*, *Escherichia coli*, *Moraxella catarrhalis*, *Candida albicans*, and *Candida tropicalis* [78]. Fungi revealed a susceptibility to both EO and Ag^+^; however, Gram-positive and Gram-negative bacteria displayed a higher susceptibility to EO and metal cations, respectively [78]. The most prominent synergy was observed in Gram-positive bacteria, probably due to the higher permeability to Ag^+^ [78], whereas Gram-negative bacteria and fungi showed, respectively, an intermediate and lower synergy due to the comparatively lower permeability of their membranes to metal cations. It is plausible that EO interfere with membrane-structure-forming pores, changing the integrity and, consequently, amplifying the penetration and antimicrobial actions of cationic silver in cells [78].

### Advantages of the Encapsulation of Essential Oils in Porous Materials

As volatile substances, EO oxidize and degrade due to light, humidity, and/or heat actions. The encapsulation of EO can amplify their physical stability, reduce their volatility, and lower the interactions with light, oxygen, humidity, and pH variations [27,31]. In addition to stabilization and protection during storage, a controlled release under the appropriate conditions is also enabled [56].

Nanoparticles enable EO to come closer to the cell surfaces and disturb the cellular membranes. Their encapsulation further enables one to reach the desired therapeutic levels for the required time, simultaneously reducing the required EO concentration and expanding the applicability of these compounds in the pharmaceutical, cosmetic, and food industries [27,31], or even in aromatherapy. This effect was observed when D-limonene, a monocyclic monoterpene, was encapsulated in niosomes, i.e., bi-layered vesicles composed of non-ionic surfactants and cholesterol [79]. The work of Hajizadeh correlated the increase in the antitumor activity against three cell lines (i.e., HepG2, A549, and MCF-7) with the improved solubility, increased internalization to the cell, and controlled release of this phytochemical [79]. An increased anticancer activity was also detected against the MCF-7 cancer cell line when *Carum carvil* extract or its main component thymoquinone was encapsulated in this type of nanostructure [80].

Essential oils have been enclosed in various encapsulation systems functioning as EODS, the most common being polymers, polysaccharides, and peptides (Table 4). These compounds are biodegradable and non-toxic, which can be favorable when applied in the food or agriculture industries [81]. However, the susceptibility to biodegradation can lead to possible alterations in the encapsulation system, EO composition, and activity over time.

Encapsulation in zeolites has the advantages of the adaptability of the crystal size and morphology, a high superficial area, stability in suspensions, greater access to the micropores, and reduced diffusion limitations [96,97]. Both the porosity and chemical composition of zeolites have influences on the size and strength of the compound’s adsorption. The encapsulation of EO or EO components in these structures is further encouraged by their good biocompatibility and low toxicity [98].

Zeolites not only offer a stable framework, specific ion exchange properties, and adsorption capacities, but their porous nature and inherent pH-sensitive properties also enable the entrapment and selective release of the encapsulated compounds [61,97,99,100,101]. This effect could be beneficial for acid tumor environments, topical applications, or textile applications. Additionally, zeolites have shown a better cellular uptake and less side effects than traditional drug delivery systems, without diminishing the desired pharmacological effects [61,97].

The attachment of different groups to the surfaces of zeolites can be applied to increase the biocompatibility and enhance the cellular uptake of the encapsulated compounds [14]. Vilaça et al. encapsulated the anticancer drug 5-fluorouracil (5-Fu) in different zeolites (NaY and LTL) and then tested cancer lines in vitro and using an in vivo model. They demonstrated that the zeolites’ surface modification with positive charges improved its uptake and improved the drug’s effectiveness, resulting in a greater tumor reduction [14]. Furthermore, surface modification could be employed to enhance the loading capacity based on the characteristics of the encapsulated compound(s) [102,103].

The conventional encapsulation methods, such as phase separation, spray drying, solvent evaporation, or high-pressure homogenization, can be inadequately used with EO, since high temperatures or pressures can alter them [84]. Other processes may be more suitable, such as gas-saturated solutions, which do not involve extreme conditions but, nonetheless, require biodegradable and non-toxic carriers or shell materials [84]. As EO encapsulation in zeolites is limited due to their high volatility, low photostability, and thermolability, high-concentration solutions are used [56]. This process is initiated by the zeolites’ drying at 160 °C to physically desorb the water molecules from the structure [56]. The adsorbed water molecules block the active sites, and their removal increases not only the pore size but also the adsorption capacity [56,68,104]. Then, the zeolites are added to an EO-concentrated solution, and the mixture is kept under agitation in a closed vessel at room temperature. If the solvent is ethanol, it can be evaporated under vacuum conditions to obtain the final product [56]. The efficiency of clinoptilolite, a natural zeolite, in the adsorption and release of oregano essential oil was studied, and a higher stability and release up to two times slower in comparison to free EO evaporation were observed [104]. An increase in eucalyptus EO retention in gaseous phase was also observed [105]. In aqueous phase, zeolites that were covalently linked with β-cyclodextrins more efficiently reduced the release of the EO than those that were not [56,96]. 

EO compounds’ retention capacity is dependent not only on the zeolites’ adsorption capability but also on their structure, as well as their physical and chemical properties. Strzemiecka et al. reported that the encapsulation of menthol, which has a 3D structure and a secondary hydroxyl group, in zeolites was easier to achieve than the encapsulation of *p*-cymene, a planar aromatic hydrocarbon [56,68]. Since menthol is a larger molecule, it might not enter the micropores. However, *p*-cymene is released more easily. On the other hand, the adsorption and desorption behavior of geranial and geraniol, a hydrogenated derivative of geranial, are related not to the structure but rather to the character of their functional groups (aldehyde and alcohol, respectively) [68]. It was observed that limonene was better retained within zeolites than linalool, which could be due to differences in the molecular sizes and to the zeolite pore size, respectively [98]. Furthermore, both molecules demonstrated a higher thermal stability and capacity for linear evaporation over time, independent of the initial quantity encapsulated [98]. The molecules’ desorption is dependent not only on their structure but also on the pore type in which they are retained and the interactions with the zeolite, namely the hydrogen bonds or London dispersion forces [68].

**Figure 6:**
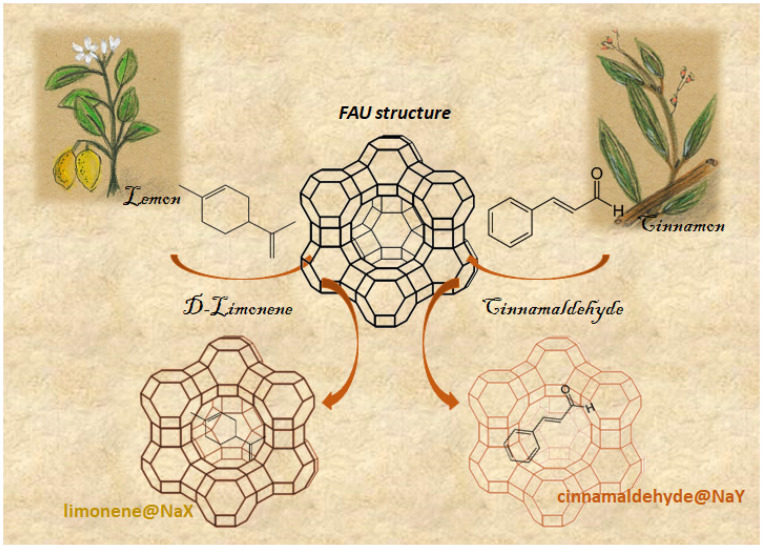
General scheme of the encapsulation of the EO compounds D-limonene and cinnamaldehyde in NaX and NaY faujasite zeolite structures, respectively (adapted from [106]).

Successful encapsulation was achieved using different aromas, including vanillin (Van), limonene (Lim), cinnamaldehyde (Cinn), and methyl anthranilate (MA). These small, organic, volatile molecules, which are present in EO, were entrapped into NaX, NaY and NaMOR zeolites [106]. Figure 6 displays a schematic representation of the different steps performed to encapsulate these EO compounds, using molecules that were both obtained by extraction from their parent EO, lemon and cinnamon, as examples. The authors studied the loading and desorption of the aroma molecules by thermogravimetric analysis (TGA). At time 0, the loading capacity of the zeolites used for the EO compounds was Cinn@NaX < Lim@NaY < MA@NaY < Van@NaMOR, with the first ones showing similar values (27 and 27.6%). The most promising carriers, MA@NaY (34.2%) and Van@NaMOR (38.2%), were followed over a longer period of time by TGA (720 days), since these systems showed the best desorption profiles, and the fragrance release mechanisms of both of them obey zero-order kinetics [106]. These results confirm that zeolite structures are ideal hosts for encapsulating several EO compounds.

EO encapsulation in zeolites can be confirmed by infrared spectroscopy (FTIR), whereas the interactions of compounds with zeolites can be established by inverted gas chromatography [106,107]. Adsorption-desorption isotherms, electron microscopy (SEM/EDX), and spectroscopy techniques (XRD, UV/Vis, XPS, ^1^H NMR, and ^13^C and ^27^Al solid-state MAS NMR and SSN-NMR) provide useful evidence regarding the encapsulation method used for entrapping organic molecules in zeolites [96,108,109,110]. Thermodynamic parameters, such as the entropy, enthalpy, and Gibbs free energy are important when evaluating physical-chemical affinity during the adsorption or desorption processes [56]. The complex mixtures of compounds with a distinct volatility in EO means that the chemical profile’s characterization is essential. The intermediate to high volatility and the intermediate to low polarity make EO ideal for gas chromatography analysis (GC) [16]. GC coupling with mass spectrometry (MS), flame ionization, or thermal conductivity detectors enables not only the identification but also the quantification of EO compounds [107]. Demirpolat and colleagues (2022) demonstrated that GC-MS analysis coupled with an FID detector can be used to determine variations in chemical properties resulting from genetic characteristics, the plant part, environmental factors, and analytical methods used [111]. Additional theoretical work was performed through frontier molecular orbital analysis, molecular electrostatic potential analysis, reduced density gradient analysis, and Fukui functions to further understand the structural characteristics and properties of, and interactions between, the main components of *Aethionema sancakense* EO [111]. 

## 4. Essential Oils Encapsulated in Zeolites 

### 4.1. Food Industry

Zeolites have been widely applied to agriculture, aquaculture, and livestock farming, specifically to supplement and remove unwanted compounds, as well as for animal residue treatment in order to reduce odors and control humidity [67]. The incorporation of essential oils into these frameworks could provide an interesting alternative to antibiotics, because they can interfere with growth without inducing microbial resistance [112]. Moreover, encapsulated EO could be used as antioxidant substitutes as well, since they possess antioxidant activity and are more resistant to environmental changes, avoiding degradation when used as animal feed additives [112].

The effect of the addition, whether individual or combined, of clinoptilolite and a mixture of carvacrol, cinnamaldehyde, and capsicum oleoresin to broiler chicken feed was studied. In both cases, positive effects were registered in the chicken’s weight gain, alongside a better antioxidant status and sensorial improvement of the meat [113]. Improvements in the yield and antioxidant effect in haying hens were verified when encapsulating *Origanum syriacum L.* EO in zeolites [112]. Additionally, the authors also demonstrated an improvement in the egg quality, lipids peroxidation, and in the oxidative stress parameters [112]. However, unwanted results were shown for these parameters at 600 mg/kg. Thus, attention should be given to the concentrations used [112]. The encapsulation of *Lavandula stoaches* EO in zeolites was linked with weight gain in broilers without additional feed consumption [114]. This could be due to the growth-promoting effect of this EO, since it is not only antioxidant and antimicrobial, resulting in a decrease in the number of pathogenic bacteria, but also stimulates digestion by increasing the oxidative stability and enhancement of immune response [114]. The positive effects of EO on the growth and health of chickens mainly result from the EO antimicrobial properties and their ability to stimulate the immune system [113]. Additionally, EO are capable of activating antioxidant enzymes, which influences the antioxidant protection effect [113]. 

Cinnamon EO is an essential oil with effective antimicrobial activity against foodborne pathogens and fungi. Niu’s work demonstrated that the encapsulation of this EO in Ag^+^/Zn^2+^-permutite, a class of artificial zeolites, and its posterior application in ethyl cellulose pads were strongly effective against *Aspergillus niger* and *Penicillum* sp., offering the controlled decay of Chinese bayberry fruit [115]. The antimicrobial activity was considered to be a result of the synergistic effect of the essential oil and Ag^+^/Zn^2+^.

With the goal of producing an eco-friendly biopesticide, clove bud EO was encapsulated in gelatin, a synthetic zeolite (LTA), and a natural zeolite (clinoptilolite) [116]. All the formulations demonstrated prolonged effects on the mortality of *Phthorimaea operculella,* a potato tuber moth, at 40 μg/L [116]. The synthetic zeolite showed the strongest antifungal activity against the grey mold *Botrytis cinerea*, even at the lowest tested EO concentration. Nonetheless, all the formulations prevented infection and disease development [116]. Ebadollahi’s work [117] also evaluated the pest-controlling activity of *Eucalyptus largiflorens* EO against *Callosobruchus maculatus.* A higher encapsulation efficiency and loading percentage, as well as a greater efficiency with respect to fumigant toxicity, were observed in this EO encapsulated in zeolite 3A when compared with the mesoporous material MCM-41 and pure EO. 

### 4.2. Textile Applications

Antimicrobial textiles are attracting increasing interest not only in regard to medical goods, in order to avoid infections and disease proliferation, but also in sports clothing, automotive fabrics, furniture, and water or air purification systems [118,119]. The textile quality can be reduced by microbial activity, resulting in color alteration, strength reduction, and the appearance of odors [120]. The antimicrobial treatment of textiles with EO encapsulated in zeolites could be applied as an anti-odor system, which controls the soaring of odor-forming bacteria while averting their major hindrance (volatility), thereby lessening the duration of the odor [118,121]. Strzemiecka’s works [56,68] demonstrated that zeolites are capable of encapsulating high quantities of citral, *p*-cymene, geraniol, and menthol in both gaseous and liquid phases. The fragrant molecule triplal was encapsulated in zeolite X, and a slow diffusion of triplal from the larger crystals and constant desorption rates associated with the zeolites’ size were observed [69]. The encapsulation of oregano EO in clinoptilolite demonstrated an efficient EO retention and release, reduction in the evaporation, and increased stability. It is worth mentioning the potential utilization of these combinations in synthetic fabrics, as well as fragrance delivery systems in laundry detergents, the release being triggered by exposure to heat or humidity [69,104]. It must be considered that zeolites’ application in textiles should not impact the wearable comfort, durability, or fabric handle [122]. In this sense, the adjustment of the zeolites’ size and improvement of the properties enabling adhesion to fibers are recommended [122].

EO use in textiles can increase the market value of the product not only due to the fragrance but also because of the antimicrobial characteristics [121]. The EO of lavender, rosemary, mint, and thyme are currently implemented in textiles due to aromatherapeutic, skin-hydrating, revitalizing, or other beneficial effects [123]. The most widely used is lavender EO, since it is calming, anti-inflammatory, and capable of treating acne, eczema, and dermatitis [123]. Aromatherapeutic textiles can be applied in body-care textiles, home textiles, household cleaning, cosmetics, or even in medicine [123]. Complementarily, the insect repellent properties of some EO, such as *Citronella genus* or *Azadirachta indica*, could be an additional benefit [121]. 

### 4.3. Healthcare 

Human skin has a complex microbiome composed of persistent resident organisms, short-term residents, and transients, whose compositions vary depending on the microenvironment [124]. For example, the axillary region is a moist microenvironment that is warm and nutritionally rich in secreted salts, proteins, steroids, lipids, and fatty acids, consequently presenting with a high microbial density. Factors such as age, gender, genetic and environmental factors, hygiene, and cosmetic use can influence the quality and quantity of secretions, as well as the microflora constitution [125].

The genera *Corynebacterium* and *Staphylococcus*, namely the species *Corynebacterium striatum*, *Corynebacterium jeikeium*, and *Staphylococcus haemolyticus*, were identified as odor producers, being the first most prominent genera in men and the second most prominent in women [119,125]. *Staphylococcus hominis*, *Cutibacterium avidum*, and *Anaerococcus* spp. can additionally affect odor formation [125,126]. Even though women have more axillary apocrine glands, in men, these are larger and more active, with the humid and nutritious environment benefiting coryneform growth and suppressing *Staphylococcus* [125]. The study of the effects of the activity of encapsulated EO on micro-organisms could enable a better understanding of the range of antimicrobial effects and the consequent influences on odor formation.

Commensal micro-organisms have important effects on skin homeostasis maintenance and human health, containing the spread of opportunistic and/or pathogenic micro-organisms, such as *Staphylococcus aureus* and *Propionibacterium acnes* [127]. *Staphylococcus epidermidis* and *Staphylococcus hominis* are predominant species in this microenvironment. Bacteria belonging to Actinobacteria, Firmicutes, and Proteobacteria phyla are detected when *Staphylococcus epidermis* is not present in dominant quantities [124]. Sampled, cloned, and sequenced bacteria from nine participants indicated that *Corynebacterium*, *Staphylococcus*, *Betaproteobacteria*, *Clostridiales*, *Lactobacillus*, *Propionibacterium*, and *Streptococcus* were the most prominent genera [128]. The disruption effects of deodorant use in resident bacteria, which can lead to the increase in microbial diversity [124], must be considered.

When considering EO encapsulation for possible application in deodorants, their activity against contaminating micro-organisms should be evaluated. It is important to study *Staphylococcus aureus*, a Gram-positive bacteria common in cutaneous microflora, *Pseudomonas aeruginosa*, a Gram-negative bacillus resistant to numerous preservatives, and *Escherichia coli*. *Candida albicans* has been used as a model of yeast that is resistant to preservatives, and *Aspergillus niger* is related to deodorant contaminations and degradation [52]. These micro-organisms are also frequent contaminants of food, sanitary devices, mucous membranes, and different surfaces [129].

Zeolite-thymol composites obtained through wet, semi-dry, and dry processes were developed and characterized [129]. The antimicrobial activity of the most promising formulation (dry approach) was evaluated against *Staphylococcus aureus*, *Escherichia coli*, *Pseudomonas aeruginosa*, and *Candida albicans* species. All the strains were completely suppressed at 75 mM thymol, except for *Pseudomonas aeruginosa*, in which a bacteriostatic effect was observed [129]. The encapsulated triplal in ion-exchanged and non-ion-exchanged zeolite X showed antibacterial as well as antifungal activities against *Escherichia coli* and *Aspergillus niger* [130].

Mallard’s work [105] tested the retention capacity of *Eucalyptus* EO in an FAU structure compared with β-cyclodextrin, verifying a higher encapsulation efficiency and lower kinetic release in the gaseous phase. Furthermore, the utilization of polycarboxylic-acid-modified zeolites improved the efficiency in the aqueous and gaseous phases, resulting in a controlled EO release [105]. These results indicate possible applications in aromatherapy, cosmetics, or medicine, considering the various benefits of this EO, such as its antimicrobial, anti-inflammatory, and antipyretic properties and non-toxicity. Since this EO exhibits antibacterial activity against *Porphyromonas gingivalis* and *Streptococcus mutant*, which cause periodontitis and other dental ailments, its use in oral hygiene products or in dental treatment equipment should be investigated [46]. 

In the biomedical field, attention should also be given to the selection of the most suitable zeolite. Erionite, scocelite, and offretite are natural fibrous zeolites which belong to the zeolite subgroups with cytotoxic properties [61]. In general, the biosafety of the zeolitic particles is associated with their structural features: the particle and pore size, shape, crystallinity, and composition [61,64]. Since the internal face of the zeolites does not interact directly with the surrounding biological environment, it does not affect the toxicity of the material [61]. The nano-sized zeolites Linde Type L (LTL) and LTA demonstrate great variability in their toxic effects based on their crystal shape and alumina content, contrary to their micro-sized counterparts, which demonstrate minimal cytotoxicity [61,110]. The cytotoxicity of 150 nano-zeolites with different structural characteristics and dosages and their effects on human cervix carcinoma cells were evaluated, and it was concluded that the zeolites composed of silica and having spherical morphology were non-toxic. However, there was a nonlinear association of the toxicity with the alumina content [131]. It was also observed that the zeolites with a cubic morphology displayed a higher toxicity to the cell line when compared with those with a spherical morphology [130]. As drug delivery systems, the work of the Neves group showed that the cytotoxicity of zeolites, as hosts, depended on the cell line used [14,110,132,133]. Three different zeolites (large, medium, and small pore sizes) were used for encapsulated 5-FU and evaluated in colorectal and breast cell cancers. All the zeolite structures were non-toxic to the cells studied [132]. However, temozolomide (TMZ) was encapsulated in NaY and NaMOR and was evaluated in glioblastoma cells. Only MOR (the mordonite structure) showed no cell toxicity effects, and it was found that TMZ encapsulated in MOR can be at least three times more effective in cell death induction than the free TMZ administered both in vitro and in vivo [133]. Even though some zeolites demonstrate an intrinsic cytotoxicity, it is broadly reported that it is beneficial to use zeolites, either alone or as encapsulation systems, for targeted cancer therapeutics, pharmaceutical delivery, healing and regeneration process improvement, and as antimicrobial agents [14,61,62,64]. 

The antimicrobial activity of encapsulated EO can be measured using several methods depending on the desired purpose (Figure S2). Careful preparation must be performed to ensure that the observed effects result from the EO and not from the preparation methods [23]. The agar diffusion method is frequently recommended when working with fastidious organisms, such as anaerobic and *Helicobacter*. However, it does not allow for the determination of MIC values [134]. Since EO are hydrophobic, the agar diffusion method can be less reliable, because it does not consider the EO volatility or agar diffusion capacity, culminating in inconstant results, fake negatives, or reduced antimicrobial activity. 

Certain micro-organisms are difficult to grow in the laboratory because they require very specific incubation conditions or have a very slow growth rate [127]. Consequently, the difficult growth of some axillary bacteria, particularly the slow-growing ones, should be considered, even if this is reflected in non-representative changes in the bacterial abundance. The antimicrobial activity against such micro-organisms can only be studied in situ using sequencing-based analysis, especially when studying the effects resulting from prolonged use [127].

Prolonged studies must be considered in order to verify the effects of components on the native flora constitution [127]. It was reported that frequent deodorant users show *Staphylococcus*-dominated communities in opposition to individuals that do not use deodorants, with *Corynebacterium* predominating [124]. Moreover, it has been suggested that the use of deodorants can reduce the abundance of cultivable bacteria, especially *Corynebacterium*, a slow-growing bacterial genus that is important in axillary odor production [135]. Studies on the effect of the prolonged use of deodorant or its alternatives, such as EO encapsulated in zeolites, require not only compromised participants but also the acceptance of possible implications for health and well-being [127]. 

This review is a significant contribution illustrating the importance of zeolite-based EODS that prevent degradation and the loss of volatile compounds and achieve a sustained release over the desired shelf life. These are crucial parameters for the application of EODS in food, textiles, and healthcare products in the industry and/or hospital environments. The most challenging aspect of EO encapsulation in zeolites appears to be the selection of the most suitable combination of the zeolite framework and the respective EO or EO component so as to enhance the encapsulation efficiency, as well the beneficial action of the EO, without inducing cytotoxicity. In this sense, future research could be performed to evaluate the impact of the encapsulation of EO in zeolites on their beneficial effects, as well on their suitability for healthcare, animal feeding, textile, or aroma therapeutic applications. Additionally, zeolite selection or deliberate modification could be performed to increase the specificity of the EO action. Nonetheless, the evaluation of zeolites and EO delivery systems’ cytotoxic effects, ensuring the lowest toxicity level possible, as well as the utilization/development of reliable and specific analytical methods, could be generally beneficial for industrial applications. 

## 5. Conclusions

Increasing consumer awareness regarding the environmental and health impacts of purchased products leads to a conscious, more natural, and sustainable life. Essentials oils (EO) are volatile secondary compounds produced by aromatic plants. Their natural nature, including their olfactory, physical-chemical, and biological characteristics, as well as their proven industrial applications, make them appealing. However, EO are physically and chemically unstable and susceptible to degradation or loss. EO encapsulation in non-toxic materials as delivery systems (EODS) protects EO from degradation and allows the EO to come closer to the target, therefore achieving the desired levels in the required timeframe, hence broadening the applicability of essential oils in the pharmaceutical, cosmetic, and food industries.

This work reviewed the importance of EO and highlighted the current progress regarding EO encapsulation in zeolites as delivery systems (EODS), providing examples of their applications in food, textiles, healthcare products, and aromatherapy.

## Figures and Tables

**Table 1:** Biological properties of EO and their industrial applications.

Plant from Which EO Was Derived	Biological Properties	Industrial Applications	References
*Matricaria camomilla*	Anti-inflammatory	Eczema, dermatitis, and pronounced topical irritation treatment	[16]
*Syzygium aromaticum*	Antibacterial	Inhibition of *Salmonella enterica* growth in lactic products	[23]
*Elettaria cardamomum*			
*Cinnamomum verum*			
*Origanum vulgare*		Inhibition and depletion of *Escherichia coli* population in food	
*Citrus sinensis*	Antifungal	Activity against pathogenic fungi in agriculture	[24]
*Syzygium aromaticum*		Inhibition of *Aspergillus flavus*, *A. parasiticus*, and *A. ochraceus* growth in food	[25]
*Cinnamomum verum*		Retardation of mold growth in food and aflatoxin production	
*Melaleuca alternifolia*	Antibacterial	Soap application against *Staphylococcus aureus*	[26]
	Insect repellent	Fly and *Tribolium castaneum* repellent	[23]
	Insecticide	Treatment of larvae and *Solenopsis invicta* infestations	[27]
*Carum carvi*	Budding suppressor	Extension of potato storage time	[28]
*Origanum compactum*	Antimicrobial	Inhibition of *Escherichia coli* growth in salads	[29]
		Preservation of poultry meat against *Escherichia coli* resistant to antibiotics used in poultry	
		Sausage preservation with natural casing	
*Origanum compactum*	Antiparasitic	Acaricidal activity against *Tetranychus urticae*	[29]
	Antimalarial	Inhibition of malaria’s causative agent, *Plasmodium falciparum*	
	Antioxidant	Increased chemical stability of, and reduction in, olive oil lipolysis	
*Illicium verum*	Antiviral	Inactivation of herpes simplex virus	[30]

**Table 2:** Mechanisms of action of some EO against bacteria (adapted from [31]).

Plant Source of EO		Target Bacteria	Mechanism of Action
Scientific Name	Common Name		
*Allium sativum*	Garlic	*Escherichia coli*	Leakage induction
*Litsea cubeba*	Mountain pepper	*Escherichia coli*	Membrane destruction
*Piper nigrum*	Black pepper	*Escherichia coli*	Cell deformation, perforation, and loss of intracellular material
*Foeniculum vulgare*	Fennel	*Shigella dysenteriae*	Membrane integrity loss
*Cuminum cyminum*	Cumin	*Bacillus cereus*	Cytoplasm changes
		*Bacillus subtilis*	
*Cinnamomum*	Cinnamon	*Escherichia coli*	Cellular membrane disruption
		*Staphylococcus aureus*	
*Dipterocarpus gracilis*	Keruing	*Bacillus cereus*	Cellular membrane disruption
		*Proteus mirabilis*	
*Ocimum gratissimum*	Basil	*Escherichia coli*	Membrane permeabilization
		*Pseudomonas aeruginosa*	
		*Staphylococcus aureus*	
*Origanum vulgare*	Oregano	*Escherichia coli*	Membrane permeabilization
		*Staphylococcus aureus*	
		*Pseudomonas aeruginosa*	
*Mentha longifolia*	Wild mint	*Escherichia coli*	Cell wall damage

**Table 3:** Synergistic and additive interactions between EO against micro-organisms (bacteria and fungi).

EO Combinations	Target Organism	Method	Interaction	References
*Origanum vulgare/* *Ocimum basilicum*	*Bacillus cereus*	*Checkerboard*	Additive	[5]
	*Eschericia coli*			
	*Pseudomonas aeruginosa*			
*Origanum vulgare/* *Origanum majorana*	*Bacillus cereus*	*Checkerboard*	Additive	
	*Eschericia coli*			
*Origanum vulgare/* *Rosmarinus officinalis*	*Bacillus cereus*	*Checkerboard*	Additive	
*Origanum vulgare/* *Rosmarinus officinalis*	*Listeria monocytogenes*	Mixture	Synergistic	
	*Yersinia enterocolitica*			
	*Aeromonas hydrophila*			
*Origanum vulgare/* *Thymus vulgaris*	*Enterobacter cloacae*	*Checkerboard*	Additive	
	*Listeria Innocua*			
	*Pseudomonas fluorescens*			
*Syzygium aromaticum/* *Rosmarinus officinalis*	*Staphylococcus epidermis*	Mixture	Additive	
	*Staphylococcus aureus*			
	*Bacillus subtilis*			
	*Eschericia coli*			
	*Proteus vulgaris*			
	*Pseudomonas aeruginosa*			
	*Candida albicans*			
	*Aspergillus niger*			
*Cinnamonum zeylancium/* *Syzygium aromaticum*	*Staphylococcus aureus*	*Checkerboard*	Synergistic	[48]
	*Listeria monocytogenes*			
	*Pseudomonas aeruginosa*			
	*Salmonella typhimurium*			
	*Aspergillus niger*			
*Oreganum vulgare/* *Thymus vulgaris*	*Aspergilus flavus*	*Checkerboard*	Synergistic	[49]
	*Aspergillus parasiticus*			
	*Penicillium chysogenum*			
	*Aspergillus niger*		Additive	
*Mentha piperita/* *Melaleuca alternifolila*	*Aspergillus niger*	*Checkerboard*	Synergistic	
*Thymus vulgaris/* *Cinnamomum verum*	*Aspergillus flavus*	*Checkerboard*	Synergistic	
*Cinnamomum verum/* *Salvia rosmarinus*	*Penicillium expansum*	*Checkerboard*	Synergistic	[50]
*Cinnamonum zeylancium/* *Syzygium aromaticum*	*Bacillus cereus*	*Checkerboard*	Additive	[48]

Note: In the checkerboard method, compounds are combined in various proportions and added to a matrix divided into sections, such as microtiter plates, to determine the fractional inhibitory concentration or the effect of the combination index of each compound. The mixture method requires a comparison between the experimental data and reference values, in which synergistic, antagonistic, or additive interactions are absent.

**Table 4:** EODS encapsulation systems.

Plant Source of EO (Scientific Name)	Encapsulation System	Results	Notes	References
*Lavandula hybrida*	Polyethylene glycol	Narrow particle size and controlled release; higher encapsulation efficiency in polyethylene glycol capsules	Encapsulation through gas-saturated solutions	[82]
	n-Octenyl succinic- -modified starch		Encapsulation through gas saturation solutions and drying	
*Artemisia arborescens*	Solid liquid nanoparticles	Increased stability of the EO	Encapsulation through high-pressure homogenization	[83]
*Eucalyptus staigeriana F.*	Cashew gum	Increased bactericidal activity against *Listeria monocytogenes*		[84]
*Thymus vulgaris*	Chitosan-benzoic acid nanogel	Increased stability, availability, and antifungal activity	*Aspergillus flavus* completely inhibited under sealed and non-sealed conditions	[85]
*Mentha piperita*	Chitosan-cinnamic acid nanogel	Reduction in MIC against *Aspergillus flavus*	Study conducted under sealed and non-sealed conditions	[86]
*Zataria multiflora*	Chitosan	Increased antifungal activity		[87]
*Satureja montana*	Cellulose nanoparticles reinforced with agar-based composites	Increased susceptibility of bacteria to the EO	*Staphylococcus aureus, Listeria monocytogenes*, and *Bacillus cereus* more susceptible than *Escherichia coli* to the nanocomposite film	[88]
*Eugenia uniflora L*.	Liposomes	EO successfully incorporated	Encapsulation through dry film hydration; Required cryoprotectors	[89]
*Peumus boldus*	Gelatin and gum arabic microcapsules	High encapsulation efficiency; increased storage time and antifungal activity	Microcapsules produced by complex coacervation	[90]
*Lippia turbinata*	Gelatin/gum arabic microcapsules	Increased antifungal activity and inhibition of seed germination		[91]
*Thymus vulgaris*	Gelatin	Reduction of MIC value	Encapsulation through complex coacervation	[92]
*Piper nigrum L.*	Hydroxypropyl-betacyclodextrin	Increased stability and antibacterial activity against *Staphylococcus aureus* and *Escherichia coli* increased by 4 times; reduction in antioxidant activity	Encapsulation through inclusion complex formation	[93]
*Melaleuca alternifolia*	Glutaraldehyde crosslinked gelatin	EO release dependent on crosslinking density, polymer wall concentration, and oil content	Microcapsules produced by simple coacervation	[94]
*Mentha x piperita*	Cyclodextrins and cross-linked cyclodextrins	Controlled release		[95]

## Data Availability

Not applicable.

## Supplementary Materials

The following are available online at https://www.mdpi.com/article/10.3390/molecules27238525/s1, **Table S1.** Mechanisms of action of some EO against fungi; **Table S2.** Synergic, additive or antagonistic interactions between EO components; **Table S3.** EO and antimicrobial drugs interactions; **Figure S1.** General scheme of the building pattern of zeolites’ 3D framework, in which silicon atoms are color blue, oxygen atoms red, aluminum atoms black and hydrogen atoms white (adapted from [18,19]; **Table S4.** Zeolites applicability and designated function (adapted from [19,20,21]); **Figure S2.** Methods used to determine antimicrobial activity of EO. References [136,137,138,139,140,141,142,143,144,145,146,147] are cited in the Supplementary Materials.
