# Supplementary material for: Essential Oils Encapsulated in Zeolite Structures as Delivery Systems (EODS): An Overview
Source: Molecules. 2022 Dec 3;27(23):8525. doi: 10.3390/molecules27238525 (PMC9740572; doi:10.3390/molecules27238525)
Supplement: Supplementary file 1 [file molecules-27-08525-s001.zip › molecules-1986620-supplementary.pdf]

molecules

Review

Essential Oils Encapsulated in Zeolite Structures as Delivery Systems (EODS): An Overview

Alexandra P. Ferreira [1,2,3], Cristina Almeida-Aguiar [2,*], Susana P. G. Costa [1,*] and Isabel C. Neves [1,3,4,*]

[1] CQUM—Centre of Chemistry, Chemistry Department, University of Minho, 4710-057 Braga, Portugal
[2] CBMA—Centre of Molecular and Environmental Biology, Department of Biology, University of Minho, 4710-057 Braga, Portugal
[3] CEB—Centre of Biological Engineering, University of Minho, 4710-057 Braga, Portugal
[4] LABBELS—Associate Laboratory, 4710-057 Braga, Guimarães, Portugal
* Correspondence: cristina.aguiar@bio.uminho.pt (C.A.-A.); spc@quimica.uminho.pt (S.P.G.C.); ineves@quimica.uminho.pt (I.C.N.)

Table S1. Mechanisms of action of some EO against fungi

Plant source of EO		Target microorganism	Mechanism of action	References
scientific name	common name			
Coriaria nepalensis	Tannery tree	*Candida albicans*	Inhibition of ergosterol biosynthesis and membrane integrity disturbance	[1]
		Candida glabrata		
		Candida parapsilosis		
		Candida krusei		
Curcuma longa	Turmeric	*Aspergillus flavus*	Inhibition of ergosterol biosynthesis	
Melaleuca alternifolia	Tea tree	*Candida albicans*	Glucose-dependent external media acidification; Permeability and membrane fluidity change	
		Candida glabrata	Glucose-dependent external media acidification	
		Saccharomyces cerevisiae		
Thymus vulgaris	Thyme	*Aspergillus flavus* *Aspergillus parasiticus*	Reduction of mycelium growth and sporulation inhibition	[2]
Origanum vulgare	Oregano			
Eucalyptus globulus	Eucalyptus	*Mucor hiemalis*	Effect on degradative and enzymatic ability	[3]
		Penicillium glabrum		
		Fusarium roseum		
Mentha piperita	Mint	*Candida albicans*	Reduction of ergosterol production, induction of intracellular acidification, morphological changes, and membrane disruption	[4]

Table S2. Synergic, additive or antagonistic interactions between EO components

Combined EO components	Microorganism	Method used to study interactions	Interaction	References
**Thymol/ Carvacrol**	*Staphylococcus aureus*	Half dilution	Additive	[5]
	Pseudomonas aeruginosa			
	Escherichia coli	*Checkerboard*	Synergism/ Additive	
	Staphylococcus aureus		Antagonism	
	Escherichia coli			
	Staphylococcus aureus	Mixture	Additive	
	Pseudomonas aeruginosa			
**Thymol/ Eugenol**	*Escherichia coli*	*Checkerboard*	Synergism	
**Carvacrol/ Eugenol**	*Escherichia coli*	*Checkerboard*	Synergism	
	Staphylococcus aureus		Antagonism	
	Bacillus cereus			
	Escherichia coli			
**Carvacrol/ Linalool**	*Listeria monocytogenes*	*Checkerboard*	Synergism	
**Cinnamaldehyde/ Carvacrol**	*Escherichia coli*	*Checkerboard*	Additive	
**Cinnamaldehyde/ Thymol**	*Escherichia coli*	*Checkerboard*	Synergism	
	Salmonella typhimurium	Mixture		
**Cinnamaldehyde/ Eugenol**	*Staphylococcus* sp.	Mixture	Additive	
	Micrococcus sp.			
	Enterobacter sp.			
**Limonene/ 1,8-cineole**	*Staphylococcus aureus*	Mixture	Synergism	
	Pseudomonas aeruginosa			
**1,8-cineole/ Aromadendrene**	Methicillin-resistant *Staphylococcus aureus*	*Checkerboard*	Additive	
	Vancomycin-resistant *Enterococci*			
	Enterococcus faecalis			
**Carvacrol/ Thymol**	*Aspergillus flavus*	Mixture	Synergism	[6]
	Aspergillus alternata			
	Penicillium sp.			
	Fusarium spp.			
**Ascaridole/ Carvacrol**	*Leishmania amazonensis*	Mixture	Synergism	[7]
**Carvacrol/ Cinnamaldehyde**	*Escherichia coli*	Mixture	Synergism	[8]
	Listeria innocua			

Note: In the checkerboard method, compounds are combined in various proportions and added to a matrix divided in sections, such as microtiter plates, to determine the fractional inhibitory concentration or the effect of the combination index of each compound. The mixture method requires the comparison between experimental data and reference values in which synergism, antagonism or additive interactions are absent.

Table S3. EO and antimicrobial drugs interactions

Microorganism	Plant source of EO (scientific name)	Antibiotic/ Antifungal	Effect	References
Staphylococcus aureus	*Foeniculum vulgare*	Cefoxitin	Enhancement of inhibition zone	[9]
		Mupirocin		
		Cotrimoxazole		
		Ciprofloxacin		
Staphylococcus aureus	*Eugenia uniflora*	Amikacin	Sinergy	[10]
Escherichia coli				
Escherichia coli	*Origanum vulgare*	Penicillin	Synergy	[11]
Trichophyton rubrum	*Melaleuca alternifolia*	Iitraconazole	Synergy and MIC reduction	[12]
		Ketoconazole		
Trichophyton rubrum	*Cinnamomum verum*	Fluconazole	Synergy	[13,14]
	Syzygium aromaticum			
	Cymbopogon martini			
	Thymus vulgaris			
Trichophyton spp.	*Allium sativum*	Ketoconazole	Synergy	[15]
Pseudomonas aeruginosa	*Prunus armeniaca*	Ciprofloxacin		
Candida albicans	*Laurus nobilis*	Fluconazole	MIC reduction	[16]
Candida glabrata				
Escherichia coli	*Mentha spicata L.*	Gentamicin	Gentamicin susceptibility amplified by 30x	[17]
Candida spp.		Fluconazole	Strong anti-candida effect	
		Ketoconazole		

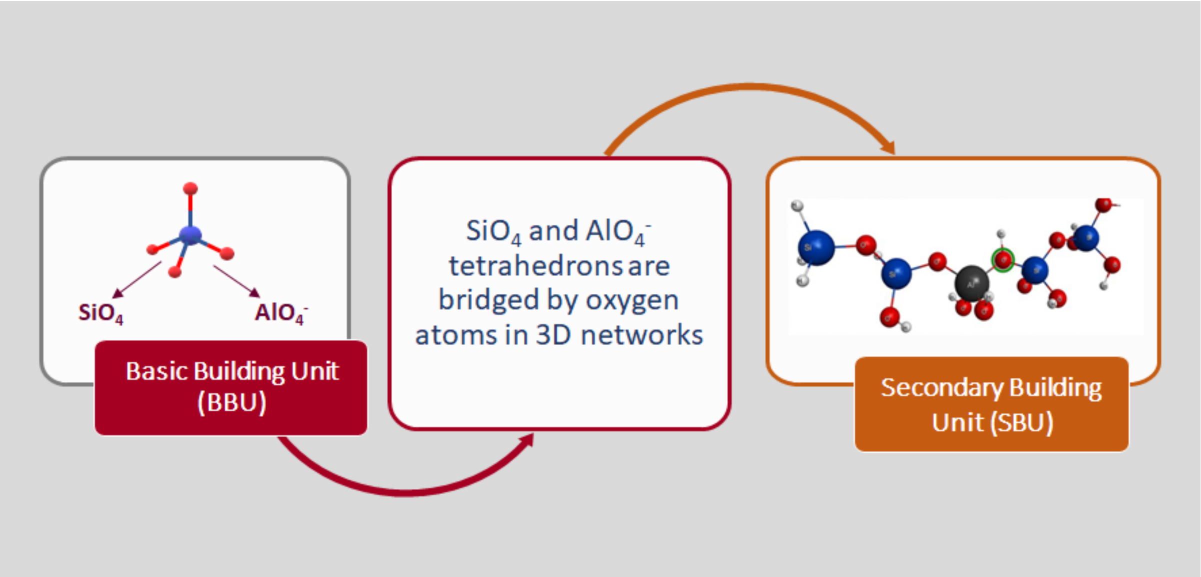

Figure S1. General scheme of the building pattern of zeolites' 3D framework, in which silicon atoms are color blue, oxygen atoms red, aluminum atoms black and hydrogen atoms white (adapted from [18,19].

Table S4. Zeolites applicability and designated function (adapted from [19–21])

Application	Function
**Construction**	Cheap construction blocks with apparent low density, high porosity and homogenous texture
	Neutralization of excessive lime produced during concrete drying
	Low weight insulating material
**Water and residual water treatment**	Ammonium extraction from municipal residual water and agriculture
	Nitrifying bacteria growth medium
	Reduction of ammonium and ammonia contents in potable water
	Cheap and selective removal of Pb2+ from potable water
**Adsorption and Catalysis**	Drying and purification of acidic gases
	Removal of water and carbon dioxide from sour natural gas
	Methane purification resulting from garbage decomposition
	Oxygen enriched air needed to hospitals, livestock and fish transport and restaurants with poor ventilation
**Nuclear ashes and residues**	Caesium and strontium removal from nuclear plants effluents
	Added to 90Sr and 137Cs contaminated soils, caused by tests or accidents, to reduce plants' uptake
	Added to pills and cookies to human consumption, in Bulgaria, to reduce intestinal absorption of Chernobyl's ashes
**Agriculture and livestock**	Poultry and swine food supplements
	Absorption of rations' contaminating aflatoxins
	Slow release of chemical fertilizers
	Soil conditioner in sandy soils and poor in clay
	Reduction of nitrate lixiviation in golf courses
	Extermination of fruit trees pests
	Zeoponic substrate
**Aquaculture**	Ammonium removal from incubator water, transports and aquariums
	Food supplements in fish feeds
**Animals' residues treatment**	Odor reduction
	Residues' humidity control
	Purification of methane gas produced
**Consumer products**	Deodorant agents for shoes, waste bins and fridges
	Water and ammonia removal from pets' urine
	Odor reduction in barns and stables
	Pillows filled with zeolites to remove odors from coffins
**Medical applications**	Hemodialysis filters to remove ammonia, allowing the reuse of the saline solution in portable dialysis systems
	Athlete's foot treatment
	Reduction of recuperation time of wounds and surgical incisions

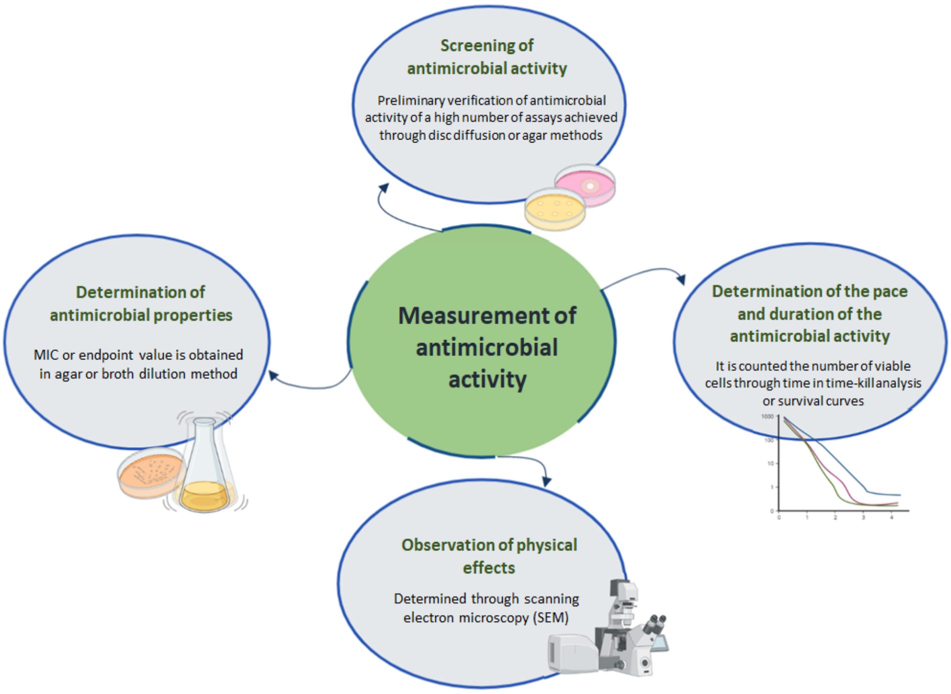

Figure S2. Methods used to determine antimicrobial activity of EO.

References

1. Chouhan, S.; Sharma, K.; Guleria, S. Antimicrobial Activity of Some Essential Oils—Present Status and Future Perspectives. *Medicines* **2017**, *4*, 58.
2. Božik, M.; Císarová, M.; Tančinová, D.; Kouřimská, L.; Hleba, L.; Klouček, P. Selected Essential Oil Vapours Inhibit Growth of Aspergillus Spp. in Oats with Improved Consumer Acceptability. *Ind. Crops Prod.* **2017**, *98*, 146–152.
3. Martins, C.; Natal-da-Luz, T.; Sousa, J.P.; Gonçalves, M.J.; Salgueiro, L.; Canhoto, C. Effects of Essential Oils from Eucalyptus Globulus Leaves on Soil Organisms Involved in Leaf Degradation. *PLoS One* **2013**, *8*.
4. Samber, N.; Khan, A.; Varma, A.; Manzoor, N. Synergistic Anti-Candidal Activity and Mode of Action of Mentha Piperita Essential Oil and Its Major Components. *Pharm. Biol.* **2015**, *53*, 1496–1504.
5. Bassolé, I.H.N.; Juliani, H.R. Essential Oils in Combination and Their Antimicrobial Properties. *Molecules* **2012**, *17*, 3989–4006.
6. Stevic, T.; Beric, T.; Savikin, K.; Sokovic, M.; Godevac, D.; Dimkic, I.; Stankovic, S. Antifungal Activity of Selected Essential Oils against Fungi Isolated from Medicinal Plant. *Ind. Crops Prod.* **2014**, *55*, 116–122.
7. Pastor, J.; García, M.; Steinbauer, S.; Setzer, W.N.; Scull, R.; Gille, L.; Monzote, L. Combinations of Ascaridole, Carvacrol, and Caryophyllene Oxide against Leishmania. *Acta Trop.* **2015**, *145*, 31–38.
8. Requena, R.; Vargas, M.; Chiralt, A. Study of the Potential Synergistic Antibacterial Activity of Essential Oil Components Using the Thiazolyl Blue Tetrazolium Bromide (MTT) Assay. *Lwt - Food Sci. Technol.* **2019**, *101*, 183–190.
9. Kwiatkowski, P.; Mnichowska-Polanowska, M.; Pruss, A.; Masiuk, H.; Dzięcioł, M.; Giedrys-Kalemba, S.; Sienkiewicz, M. The Effect of Fennel Essential Oil in Combination with Antibiotics on Staphylococcus Aureus Strains Isolated from Carriers. *Burns* **2017**, *43*, 1544–1551.
10. Pereira, N.L.F.; Aquino, P.E.A.; Júnior, J.G.A.S.; Cristo, J.S.; Vieira Filho, M.A.; Moura, F.F.; Ferreira, N.M.N.; Silva, M.K.N.; Nascimento, E.M.; Correia, F.M.A.; et al. Antibacterial Activity and Antibiotic Modulating Potential of the Essential Oil Obtained from Eugenia Jambolana in Association with Led Lights. *J. Photochem. Photobiol. B Biol.* **2017**, *174*, 144–149.
11. Gallucci, N.; Casero, C.; Oliva, M.; Zygadlo, J.; Demo, M. Interaction between Terpenes and Penicillin on Bacterial Strains Resistant to Beta- Lactam Antibiotics. *J. Appl. Microbiol.* **2006**, *10*, 30–32.
12. Roana, J.; Mandras, N.; Scalas, D.; Campagna, P.; Tullio, V. Antifungal Activity of Melaleuca Alternifolia Essential Oil (TTO)

and Its Synergy with Itraconazole or Ketoconazole against Trichophyton Rubrum. *Molecules* **2021**, *26*, 461.

13. Khan, M.S.A.; Ahmad, I.; Cameotra, S.S. Carum Copticum and Thymus Vulgaris Oils Inhibit Virulence in Trichophyton Rubrum and Aspergillus Spp. *Brazilian J. Microbiol.* **2014**, *45*, 523–531.
14. Khan, M.S.A.; Ahmad, I. Antifungal Activity of Essential Oils and Their Synergy with Fluconazole against Drug-Resistant Strains of Aspergillus Fumigatus and Trichophyton Rubrum. *Appl. Microbiol. Biotechnol.* **2011**, *90*, 1083–1094.
15. Pyun, M.S.; Shin, S. Antifungal Effects of the Volatile Oils from Allium Plants against Trichophyton Species and Synergism of the Oils with Ketoconazole. *Phytomedicine* **2006**, *13*, 394–400.
16. Nafis, A.; Kasrati, A.; Jamali, C.A.; Custódio, L.; Vitalini, S.; Iriti, M.; Hassani, L. A Comparative Study of the in Vitro Antimicrobial and Synergistic Effect of Essential Oils from Laurus Nobilis l. and Prunus Armeniaca l. from Morocco with Antimicrobial Drugs: New Approach for Health Promoting Products. *Antibiotics* **2020**, *9*.
17. Wińska, K.; Mączka, W.; Łyczko, J.; Grabarczyk, M.; Czubaszek, A.; Szumny, A. Essential Oils as Antimicrobial Agents—Myth or Real Alternative? *Molecules* **2019**, *24*, 1–21.
18. McCusker, L.B.; Baerlocher, C. Zeolite Structures. In *Studies in Surface Science and Catalysis*; 2001; Vol. 137, pp. 37–67.
19. Sels, B.; Kustov, L. *Zeolites and Zeolite-like Materials*; first edit.; Elsevier, 2016; ISBN 9780444635068.
20. Derakhshankhah, H.; Jafari, S.; Sarvari, S.; Barzegari, E.; Moakedi, F.; Ghorbani, M.; Varnamkhasti, B.S.; Jaymand, M.; Izadi, Z.; Tayebi, L. Biomedical Applications of Zeolitic Nanoparticles, with an Emphasis on Medical Interventions. *Int. J. Nanomedicine* **2020**, *15*, 363–386.
21. Mumpton, F.A. La Roca Magica: Uses of Natural Zeolites in Agriculture and Industry. *Proc. Natl. Acad. Sci. U. S. A.* **1999**, *96*, 3463–3470.
